# Circulating miR-29c-3p is downregulated in patients with acromegaly

**DOI:** 10.3906/sag-2010-245

**Published:** 2021-08-30

**Authors:** Hakan KORKMAZ, Kuyaş HEKİMLER ÖZTÜRK, Bora TORUS

**Affiliations:** 1 Division of Endocrinology, Department of Internal Medicine, Faculty of Medicine, Süleyman Demirel University, Isparta Turkey; 2 Department of Medical Genetics, Faculty of Medicine, Süleyman Demirel University, Isparta Turkey; 3 Department of Internal Medicine, Banaz State Hospital, Uşak Turkey

**Keywords:** Acromegaly, miR-29c-3p, miR-31-5p, miR-18a-5p

## Abstract

**Background/aim:**

miRNAs control various biological functions, such as cell proliferation, differentiation, signaling pathways, apoptosis and metabolism. Recently, it has been shown that there is a relationship between changes in miRNA expression and the development of acromegaly. Studies are needed to identify new disease-specific miRNAs. The aim of the current study is to evaluate plasma miR-29c-3p, miR-31-5p and miR-18a-5p steady-state levels in acromegaly. Another aim is to investigate whether there is a difference in the levels of these miRNAs in patients with inadequate control and controlled acromegaly with somatostatin analog (SSA) therapy. These miRNAs targeting the IGF-1 gene were determined by in silico estimation.

**Materials and methods:**

The study included 30 healthy controls (HC) and 20 patients with acromegaly. Anterior pituitary functions and disease activities of patients with acromegaly were evaluated at the time of study. The miR-29c-3p, miR-31-5p and miR-18a-5p levels were measured using quantitative real-time PCR (RT-qPCR).

**Results:**

The expression level of miR-29c-3p was significantly lower in patients with acromegaly compared to the HC group (p* < *0.001). This downregulation was more pronounced in patients with inadequately controlled acromegaly than in patients with acromegaly controlled with somatostatin analogues (SSA) therapy (p* = *0.016). Univariate logistic regression analysis results showed that down regulation of miR-29c-3p expression increases the risk of developing acromegaly [OR (95% Cl) *= *1.605 (1.142-2.257), p* = *0.006]. There was no significant difference between the groups in terms of miR-31-5p and miR-18a-5p expression levels (p = 0.375 and p *= *0.649, respectively).

**Conclusion:**

Plasma miR-29c-3p expression level is downregulated in patients with acromegaly, and this is more pronounced in patients with inadequate control.

## 1. Introduction

Acromegaly is an endocrine disease that usually originates from the somatotropic cells of the pituitary gland and causes the release of excess growth hormone (GH) and insulin-like growth factor 1 (IGF-1). Increased IGF-1 levels stimulate cell proliferation and inhibit apoptosis [1].

Increasing evidence has shown that miRNAs play a role in the pathophysiology of GH-secreting pituitary adenoma. Studies found that GH-secreting pituitary adenoma samples have downregulations or upregulations in the expression of various miRNAs compared to normal pituitary tissues, and some of them are associated with proliferation, migration and invasion of tumor cells [2,3]. miRNAs are used as potential circulating biomarkers for the diagnosis and prognosis of many diseases due to their stable structure and detectability in plasma [4]. GH and IGF-1 levels are used as biomarkers in the diagnosis and follow-up of acromegaly [5].

It is thought that circulating miRNAs may be useful in predicting the prognosis of patients with acromegaly and their response to treatment with somatostatin analogs (SSAs) [3,6]. New studies are needed to identify specific circulating miRNAs for acromegaly disease.

In this study, miRNAs targeting the IGF-1 gene were determined by in silico estimation. For this purpose, miRDB (http://mirdb.org/index.html) and TargetSan Release 7.2 (http://www.targetscan.org/vert_72/) algorithms were used and miR-31-5p, miR-18a-5p and miR-29c-3p were determined as candidate miRNAs. miR-29c-3p and miR-31 are tumor suppressor miRNAs. Downregulations of these miRNAs were associated with development and poor prognosis of various tumors [7–10]. Different tumor studies have shown that miR-18a has both oncogenic and tumor suppressive roles. It was thought that the dual functional of miR-18a may be attributed to fundamental differences in tumorigenic mechanisms [11–13]. These miRNAs, which have been proven to be associated with various malignancies, were not evaluated in patients with acromegaly to date.

The primary aim of the current study is to evaluate whether there is a difference in plasma miR-29c-3p, miR-31-5p and miR-18a-5p expression levels in patients with acromegaly compared to healthy controls (HCs). The second aim is to compare these miRNAs in patients with inadequately controlled acromegaly and acromegaly controlled with SSA therapy.

## 2. Material and methods

This study was conducted according to the principles of the Helsinki Declaration after it was approved by the local ethics committee. All participants were informed about the research protocol, and they declared their voluntary attendance by signed written consent. 

### 2.1. Selection of samples

Twenty patients with acromegaly (mean age 53.15 ± 11.5 years, 10 females, 10 males) and 30 healthy controls (mean age 54.5 ± 8 years, 13 females and 17 males) were included in the study. In patients with acromegaly, those with infectious diseases, rheumatic diseases, pulmonary diseases, liver failure, kidney failure, pregnancy, and different malignancy apart from acromegaly were excluded from the study. Age and sex matched HC group was randomly selected from people without any systemic disease and drug use.

Disease durations, medical history (medical, surgical, and surgery), treatment response evaluations, demographic features, comorbid conditions, treatment histories (surgery, radiotherapy, pharmacological therapy), pituitary functions, and medical histories were recorded. Controlled acromegaly was defined as random GH level, which was below 1.0 ng/mL, and IGF-1 values were in the reference range for age and sex. Inadequately controlled acromegaly was defined as mean GH >2.5 μg/L and IGF-1 >1.3 times the sex- and age-adjusted upper limit of normal (ULN). In this study, 7 of the patients with acromegaly consisted of inadequately controlled patients.

All of the patients included in the study underwent transsphenoidal pituitary surgery and were still receiving SSA therapy. There were no patients who received SSA treatment before transsphenoidal pituitary surgery. Eight patients required treatment for some degree of hypopituitarism, namely with L-thyroxine, hydrocortisone and/or gonadal steroids. All patients with hypothyroidism were receiving adequate hormone replacement. 

### 2.2. Biochemical analysis

After at least 8 h of fasting, 5 cc peripheral venous blood samples were collected into three blood collection tubes (2 EDTA-containing and 1 gel-included biochemistry tubes) from all participants.

Serum fasting blood glucose (FBG), alanine aminotransaminase (ALT), creatinine, uric acid, total cholesterol (TC), triglyceride (TG), and high-density lipoprotein cholesterol (HDL-C) were measured using a Beckman Coulter AU 5800 chemistry analyzer (Beckman Coulter, Brea, CA, USA). Low-density lipoprotein cholesterol (LDL-C) was calculated with the Friedewal formula [LDL-C= TC - (HDL-C+ (TG/5)].

Serum IGF-1 and GH levels were measured by electro-chemiluminescence immunoassay (Elecsys IGF-1; Roche Diagnostics, Mannheim, Germany). The normal range of serum IGF-1 was evaluated by sex and age. Age and sex-normalized levels of IGF-1, rare GH< 0.4 ng/mL in oral glucose tolerance test (OGTT) after surgery and random GH<1 ng/mL when treating with SSA were accepted criteria for cure or good biochemical control [14].

### 2.3. miRNA extraction, primer design 

Extraction of microRNAs was carried out using a peripheral blood plasma sample with the Hybrid-RTM miRNA isolation kit (GeneAll Biotechnology, Korea). The purity and concentration of these microRNAs were measured using an ultraviolet spectrophotometer at 260 nm and 280 nm absorption using a Nanodrop spectrophotometer Thermo Fisher Qubit 3.0 (Thermo Fisher Scientific Inc.,Waltham, MA, USA). The microRNA was then stored at –80 °C up to the cDNA synthesis stage. NCBI (National Center for Biotechnology Information) nucleotide database was used for primer sequences of miRNAs. 

The sequence used for miR-29c-3p includes primers as follows: Forward: 5’- TAGCACCATTTGAAATCGGTTA-3´(Accession No: MIMAT0000681). For miR-31-5p: Forward: 5’-AGGCAAGATGCTGGCATAGCT-3’(Accession No: MIMAT0000089). For miR-18a-5p: 5’-TAAGGTGCATCTAGTGCAGATAG-3’(Accession No: MIMAT0000072), while U6 snRNA as housekeeping gene includes primers as follows: Forward: 5’- GCTTCGGCAGCACATATACTAAAAT -3´. 

### 2.4. cDNA synthesis and RT-PCR measurement

Complementary DNA (cDNA) was obtained from the isolated miRNAs using the HyperScriptTM Reverse Transcriptase kit (GeneAll Biotechnology, Korea). Reverse transcription was performed using SimpliAmp Thermal Cycler (Waltham, MA, USA). Quantitative real-time PCR reactions (qRT-PCR) were performed with the high-capacity StepOnePlus real-time PCR System (Waltham, MA, USA). The thermal cycling conditions were as follows: an initial denaturation step at 95 ℃ for 10 min, 40 cycles of PCR amplification at 95℃ for 15 s, and 60℃ for 1 min, followed by a melting curve analysis program according to the instrument documentation. All real-time PCR reactions were run in triplicate. The sequences of all the primers used are listed in Table 1. The expression levels of miR-29c, miR-31, and miR-18a were examined using the real-time PCR technique and the SYBER Green method, using U6 snRNA as an internal control (housekeeping gene). The cycle threshold (CT) of investigated primers was determined and normalized to the housekeeping gene, RNU6. Fold change of each miRNA expression was calculated using the equation 2^−ΔΔCt^. 

**Table 1:** Demographic and clinical characteristics of patients with acromegaly and HCs.

	Acromegaly	Healthy control
Sex (male/female)	10/10	17/13
Age (years)	53.15 ± 11.5	54.5 ± 8
Duration of diagnosis (years)	6 ± 3	
Somatostatin analog theraphy	n = 20	
Inadequately controlled acromegaly	7/20	
Contrelled acromegaly	13/20	
Postoperative radiotheraphy	n = 1	
Hypopituitarism	n = 4	
ACTH deficiency	1/4	
Gonodotropin deficiency	3/4	
TSH deficiency	3/4	

### 2.5. Statistical analysis 

SPSS for Windows version 22.0 (IBM Corp., Armonk, NY, USA) was used for statistical analysis. For the comparison of categorical data, a chi-square test (sex) was used. After the Kolmogorov–Smirnov test, the normal distribution parameters (age, HDL-C, LDL-C and TC) were compared with the Student’s t test, and parameters with non-normal distribution were compared using the Mann–Whitney U test. miR-29c-3p expression levels of inadequately controlled and controlled acromegaly patients with SSA therapy were compared by using the Mann–Whitney U test. To evaluate the relationship between parameters, Pearson correlation analysis was used for those with parametric distribution, and Spearman correlation analysis was used for those with non-parametric distribution. The effect of miR-29c-3p expression on acromegaly development was evaluated by univariate logistic regression analysis. The p value *<*0.05 was considered statistically significant.

## 3. Results

The clinical and demographic characteristics of the groups are shown in Table 1. There was no significant difference in age and sex between HC and acromegaly groups. (p = 0.820 and p = 0.643, respectively). Laboratory features of the groups are shown in Table 2. Fasting plasma glucose (FBG) level was higher in patients with acromegaly than in the HC group (p *< *0.001). There was no difference between the groups in terms of creatinine, alanine aminotransferase (ALT) and lipid levels (for each, p > 0.05).

**Table 2:** Biochemical characteristics of of the groups.

	Acromegaly	Healthy control	p
FBG (mg/dL)	113±38	93±11	<0.001
Creatinine (mg/dL)	0.97±0.19	0.83±0.25	0.086
ALT (U/L)	16±7	17±10	0.475
TC (mg/dL)*	198.5 (121-256)	2094 (151-293)	0.081
TG (mg/dL)	114±60	112±79	0.677
HDL-C (mg/dL)*	50 (34-67)	48.50 (31-85)	0.272
LDL-C (mg/dL)*	120 (76-189)	130.5 (86-183)	0.096
IGF-1 (ng/mL)	228.5±193	-	
GH (ng/mL)	0.98±1.72	-	

There was no significant difference between the groups in terms of miR-31-5p and miR-18a expression levels (p = 0.375 and p = 0.649, respectively).

**Figure 1:**
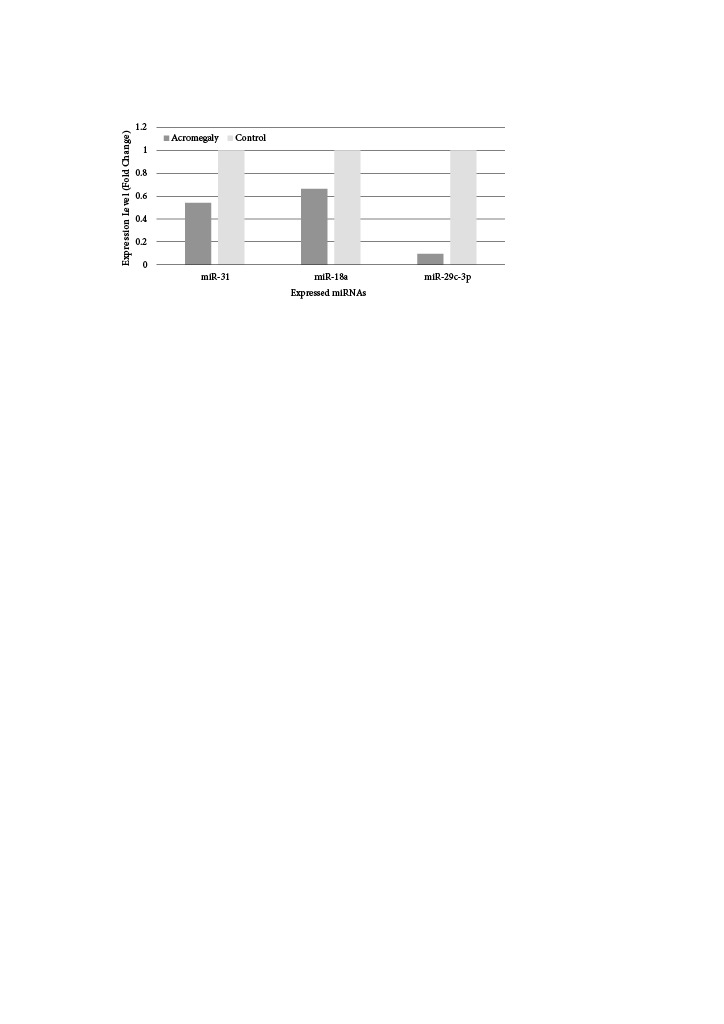
Fold changes of miRNAs in patients with acromegaly and HCs.

In patients with acromegaly, miR-29c-3p expression was significantly downregulated compared to the HC group (p < 0.001). Univariate logistic regression analysis results demonstrated an important association between acromegaly development and downregulation of miR-29c-3p expression [OR (95% Cl) = 1.605 (1.142–2.257), p *= *0.006]. Average expression levels and fold changes of miRNAs isolated from plasma are given in Figure 1 and Table 2. 

**Figure 2:**
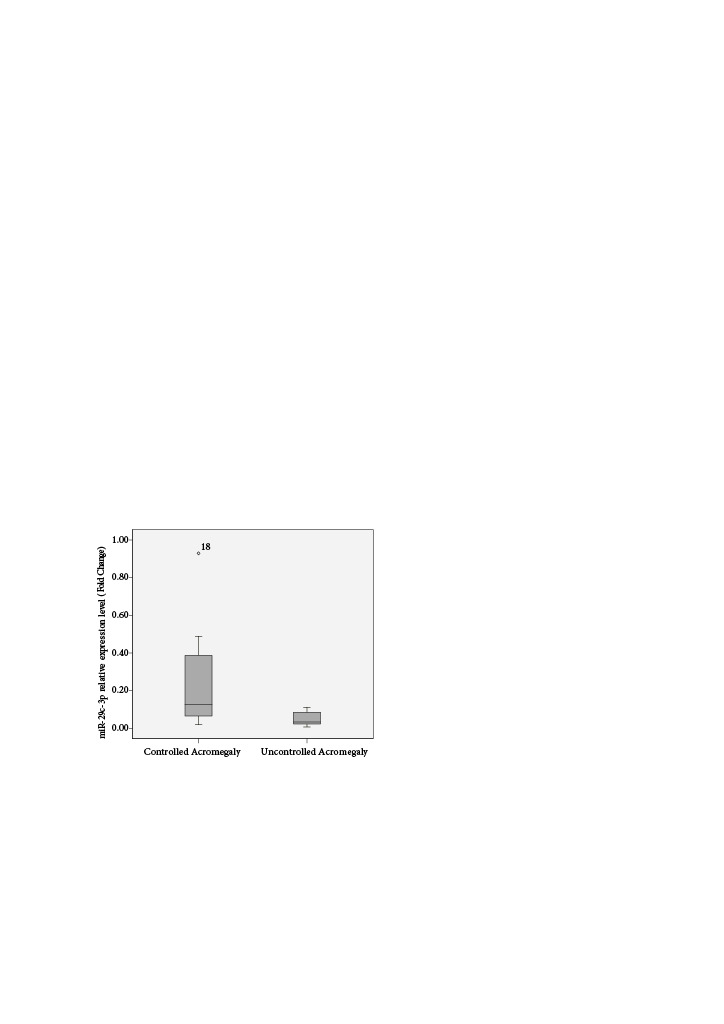
Fold changes of miR-29c-3p in patients with controlled and uncontrolled acromegaly.

miR-29c-3p expression was downregulated in patients with inadequately controlled acromegaly compared to those controlled with SSA therapy (p = 0.016, Figure 2). There was no significant difference in miR-18a-5p and miR-31-5p expression levels between patients with inadequately controlled and controlled acromegaly with SSA therapy (p = 0.427 and p *= *0.571, respectively).

There was no correlation between plasma miR-29c-3p and IGF-1 levels in patients with acromegaly.

## 4. Discussion

In the current study, the miR-29c-3p level was found to be significantly lower in patients with acromegaly compared to age- and sex-matched HC. In addition, the miR-29c-3p expression level was lower in patients with inadequately controlled acromegaly compared to patients controlled with SSA therapy. To the best of our knowledge, this is the first study to evaluate plasma miR-29c-3p expression levels in patients with acromegaly.

There are limited number of studies evaluating circulating miRNAs in patients with acromegaly. Valassi et al. showed that changes in plasma miR-103a-3p and miR-660-5p expressions were found in patients with acromegaly, and these changes were related to both structural and biochemical parameters of bone metabolism [15]. Zhao et al. showed that exosome miR-423-5p and miR-320a expression were lower in GH-secreting pituitary adenoma than in normal pituitary tissue. They found that miR-423-5p inhibits cell proliferation and migration, induces cell apoptosis, and reduces GH release during an in-vitro study. Researchers suggested that miR-423-5p plays a central role in promoting tumor formation in somatotroph adenomas and may act as a potential biomarker for therapeutic interventions using gene therapy [16]. Circulating miRNAs can be a potentially useful diagnostic marker by improving the classification of acromegaly. Therefore, new circulating miRNAs specific to acromegaly should be identified.

In the current study, plasma miR-29c-3p expression was evaluated in patients with acromegaly. miR-29c-3p is a tumor suppressor and was found to be downregulated in many malignant diseases. In addition, downregulation of miR-29c-3p expression was shown to be associated with lymph node metastasis, poorly differentiated tumor, advanced TNM stage and poor prognosis [7,9,17]. In this study, plasma miR-29c-3p expression was significantly lower in patients with acromegaly compared to HCs. The roles of miRNAs in the development of acromegaly were evaluated by logistic regression analysis. The downregulation of plasma miR-29c-3p was shown to increase the risk of acromegaly 1.6 times.

Transsphenoidal pituitary surgery is preferred for first-line treatment in patients with acromegaly. Since the remission rate of the disease after pituitary surgery is 28%–39%, most of the patients with acromegaly need additional treatment such as radiosurgery and medical treatment. SSAs are widely used in the medical treatment of acromegaly, but patient response to this treatment is highly variable [18–21]. Predicting the medical treatment response will be useful for determining the treatment plan [3]. Therefore, there is a need to identify biomarkers that may have a role in predicting treatment responses. Mao et al. evaluated the relationship between response to SSA and miRNAs expression levels after administering SSA therapy to patients with GH secreting adenoma for 4 months before the pituitary operation. They found that miR-524-5p was downregulated patients who were SSA responders compared to SSA nonresponders, and miR-524-5p was upregulated after SSA treatment was discontinued. Researchers suggested that determining target miRNAs can be important in predicting the response to SSA therapy in acromegaly [3]. In the current study, miR-29c-3p expression was lower in patients with inadequately controlled acromegaly than in patients controlled with SSA therapy. miR-29c-3p may be an alternative biomarker for predicting the SSA treatment response of patients with acromegaly. However, whether miR-29c-3p had any role in predicting the response to SSA therapy can be understood with prospective studies in which more cases are included. Such a possible relationship may be due to several reasons. miR-2pc-3p is a miRNA that targets the IGF-1 gene (based on in- silico evaluation) and may show effects at the hepatic level. In addition, increased SST2 expression, dense tumor granularity and low proliferative index (Ki-67) expression in tumor tissue are histopathological findings showing that the response to SSA treatment will be better [22]. miR-29c-3p expression levels may also be associated with these histopathological findings. There is a need for advanced molecular studies at tissue level on this subject.

This study has some limitations. The first is that it is a cross-sectional study, so no information about causality can be provided. Secondly, the number of samples is relatively low. However, it is difficult to reach large study groups as acromegaly is a rare disease. In addition, the fact that this study did not evaluate the histopathological findings in tumor tissue and the relationships between these miRNAs is another limitation.

In conclusion, expression of miR-29c-3p level is downregulated in patients with acromegaly compared to healthy adults. This downregulation is more pronounced in patients with acromegaly inadequately controlled with SAA therapy. These results suggest that miR-29c-3p may be a target miRNA for acromegaly. We believe that this study will shed light on future studies.

## Informed consent

This study was approved by the local ethics committee (13.05.2020, decision number:139). All patients to be included in the study were informed verbally and in writing about the research and informed consent forms were obtained from those who agreed to participate in the study.

## Funding

This study did not receive any financial support.
